# Gestational diabetes mellitus, pre-pregnancy body mass index, and gestational weight gain as risk factors for increased fat mass in Brazilian newborns

**DOI:** 10.1371/journal.pone.0221971

**Published:** 2019-08-29

**Authors:** Laísa R. S. Abreu, Meghan K. Shirley, Natália P. Castro, Verônica V. Euclydes, Denise P. Bergamaschi, Liania A. Luzia, Ana M. Cruz, Patrícia H. C. Rondó

**Affiliations:** 1 Nutrition Department, School of Public Health, University of São Paulo, São Paulo, SP, Brazil; 2 Epidemiology Department, School of Public Health, University of São Paulo, São Paulo, SP, Brazil; 3 Geraldo de Paula Souza Health Center, School of Public Health, University of São Paulo, São Paulo, SP, Brazil; Monash University, AUSTRALIA

## Abstract

**Background:**

Gestational diabetes mellitus (GDM) is a common complication of pregnancy. It may predispose offspring to increased fat mass (FM) and the development of obesity, however few data from Latin America exist.

**Objective:**

To investigate the influence of GDM on newborn FM in mother-newborn pairs recruited from a public maternity care center in São Paulo, Brazil.

**Methods:**

Data were collected cross-sectionally in 2013–2014 from 72 mothers diagnosed with GDM, and 211 mothers with normal glucose tolerance (NGT). Newborn FM was evaluated by air-displacement plethysmography (PEA POD), and relevant demographic and obstetric data were collected from hospital records. Associations between maternal GDM status and newborn FM were investigated by multiple linear regression analysis, with adjustment for maternal age, pre-pregnancy BMI, gestational weight gain, type of delivery, sex of the child, and gestational age.

**Results:**

FM was greater in GDM versus NGT newborns in a bivariable model (Median (IQR), GDM: 0.35 (0.3) kg vs. NGT: 0.27 (0.2) kg, *p* = 0.02), however GDM status was not a significant predictor of FM with adjustment for other variables. Rather, pre-pregnancy BMI (coefficient (β) 1.46; 95% confidence interval (CI) 0.66, 2.27), gestational weight gain (β 1.32; 95% CI 0.49, 2.15), and male sex (β -17.8; 95% CI -27.2, -8.29) predicted newborn FM. Analyzing GDM and NGT groups separately, pre-pregnancy BMI (β 6.75; 95% CI 2.36, 11.1) and gestational weight gain (β 5.64; 95% CI 1.16, 10.1) predicted FM in the GDM group, while male sex alone predicted FM in the NGT group (β -12.3; 95% CI -18.3, -6.34).

**Conclusions:**

Combined model results suggest that in our cohort, pre-pregnancy BMI and gestational weight gain are more important risk factors for increased neonatal FM than GDM. However, group-specific model results suggest that GDM status may contribute to variation in the relationship between maternal/offspring factors and FM. Our use of a binary GDM variable in the combined model may have precluded clearer results on this point. Prospective cohort studies including data on maternal pre-pregnancy BMI, GWG, and glycemic profile are needed to better understand associations among these variables and their relative influence on offspring FM.

## Introduction

In 2017, Brazil was ranked first in South and Central America and fourth worldwide among countries with the highest prevalence of adult diabetes [1]. In the same year, globally, an estimated 21.3 million births were affected by hyperglycemia in pregnancy [1]. Gestational diabetes mellitus (GDM) accounted for approximately 86.4% of these hyperglycemic pregnancies. The clinical and public health relevance of GDM is increasingly recognized as it has become one of the most common complications of pregnancy, although prevalence estimates vary by country, region, and diagnostic criteria [2–4].

GDM is characterized by the development of glucose intolerance in pregnancy [5], and it increases the risk of adverse health outcomes for mothers and their offspring if not well controlled [6, 7]. The global pandemic of obesity and overweight [8] contributes to the increased risk of hyperglycemia in women of childbearing age [9–11]. Notably, countries with the highest rates of obesity demonstrate the highest rates of diabetes [12]. GDM is typically diagnosed by oral glucose tolerance test at 24–28 weeks of gestation, and often resolves after birth. However, women who have had GDM are at higher risk of developing GDM in subsequent pregnancies, and more likely to develop overt type 2 diabetes mellitus and cardiovascular disease [13–15]. Women facing these diagnoses in the poorest areas of low- and middle-income countries are more vulnerable to adverse reproductive outcomes associated with limited access to care [12]. Ninety percent of cases of hyperglycemia in pregnancy are estimated to occur in such areas [12].

Fetal exposure to the altered metabolic intrauterine environment associated with GDM is suggested to increase fetal fat mass (FM) and affect fetal growth and development, thus increasing the risk of obesity and type 2 diabetes later in life [16–18]. Several studies have assessed the relationship between GDM and newborn FM [16, 19–24], and many, but not all, have reported positive associations (see Discussion). However, to our knowledge, no such study has been conducted in Latin America. Investigations across populations are important, as geography and ethnicity are associated with variation in body composition [25–27], and ethnicity is also a recognized risk factor for GDM [28]. The aim of this study was to test the influence of GDM, controlling for other maternal and neonatal factors, on the FM of newborns from a large Brazilian city.

## Participants and methods

This cross-sectional study carried out in 2013–2014 sought to recruit mothers with GDM or normal glucose tolerance (NGT) from a large, public maternity care center in São Paulo, Brazil. Participants were selected based on information included in their medical records and antenatal cards. Exclusion criteria were: age < 18 years; multiple pregnancy; hypertension; hormonal disorders; chronic infectious disease; smoking or drug use; alcohol consumption in pregnancy; delivery at < 37 weeks or ≥ 42 weeks’ gestation; birthweight < 2,500 g; neonatal intensive care unit admission > 2 days; and genetic disorders of the newborn.

The following steps (recruitment and all maternal/neonatal measurements) were performed within 24–72 hours of delivery. Mothers were invited to participate and answered questions to assess demographic, socioeconomic and obstetric factors. They reported their weight prior to pregnancy, which was compared against first-trimester weight recorded in the antenatal card. Recorded first-trimester weight, and height measured with a Tonelli Model 120A stadiometer (Tonelli, Criciúma, SC, Brazil) to the nearest 0.1 cm [29] were used to calculate pre-pregnancy body mass index (ppBMI) in kg/m^2^, following previous authors [30, 31]. Data on maternal date of birth, parity, newborn date and time of birth, type of delivery, sex of the newborn, gestational age, birth weight, birth length, and newborn head and chest circumference at birth were collected from hospital records. Gestational age was determined by ultrasonography performed up to 20 weeks’ gestation. Gestational weight gain (GWG) was defined as the difference between maternal weight recorded at the first antenatal visit and maternal weight measured in the week prior to delivery.

Newborn length was measured to the nearest 0.1 cm with a Seca 416 Infantometer (Seca, Hamburg, Germany). Chest, head and abdominal circumferences of the newborn were obtained with a Seca non-stretchable tape to the nearest 0.1 cm. Newborn weight and body composition were assessed by air-displacement plethysmography (ADP) using the PEA POD system (COSMED USA, Inc., Concord, CA). ADP relies on densitometric principles to generate a two-component body composition model comprised of FM and fat-free mass (FFM). Specifically designed to measure infants, the PEA POD has been validated against reference deuterium isotope dilution and the three- and four-component models [32–34]. Ma et al. [32] reported excellent within- and between-day test-retest reliability, although a limitation of the method is its lack of accounting for variation in FFM density and hydration beyond age and sex differences. PEA POD measurements are quick, easy and harmless, and thus widely used to assess the body composition of infants weighing up to 8 kg.

Infants were placed within the PEA POD’s temperature-controlled measurement chamber for 2–3 minutes, where they remained visible to the mother and operator through a plexiglass window. Infants were nude and those with hair had a small amount of baby oil and a thin cap applied to the head, in order to avoid air pockets created by hair and clothing. Measured body volume was corrected for thoracic gas volume and surface area artefact, which is warmer, more compressible air close to the skin’s surface. Measured body mass was divided by corrected body volume to derive total body density, which, along with assumed constant density values for FM and FFM, can be used to calculate %FM. The density value 0.9 g/mL was used for FM, and values reported by Fomon et al. [35] were used for FFM: 1.064 g/mL for girls at birth, and 1.063 g/mL for boys. FM and FFM were calculated from %FM using body mass.

### Statistical analysis

Stata version 11 software (Stata Corporation, College Station, TX, USA) and the R language for statistical computing (version 3.6.0) [36] in RStudio (version 1.1.463) were used for statistical analysis. All variables of interest were evaluated for missing observations, potential outliers, and normality. Mother’s age, ppBMI, GWG, *per capita* household income, birth weight, gestational age, FM and abdominal circumference were highly positively skewed. Log transformation was attempted, however the variables remained significant under Shapiro-Wilk. Several of the variables contained outliers which contributed to their skew, including GWG, *per capita* household income, birth weight, gestational age, FM and abdominal circumference. Sensitivity analyses performed with outliers removed yielded unchanged results.

Chi-squared, Mann-Whitney-Wilcoxon, independent sample t-tests, and one-way ANOVA with Tukey post-hoc adjustment were used where appropriate to test for differences in maternal age, height, parity, delivery type, ppBMI, GWG, work status (yes or no), and *per capita* household income between GDM and NGT groups. Tests were similarly carried out for differences between the groups for infant sex, birth weight, gestational age, FM, FFM, length at birth, and circumferences of the head, chest and abdomen.

A number of prior studies have used FM adjusted for weight (percentage fat, or %FM) to assess infant FM [37–39], however others have criticized this practice as statistically flawed [40]. We followed Wells and Victora [40] in adjusting FM for FFM, rather than weight, using log-log regression. Specifically, FM and FFM were natural log transformed, with logFM then regressed on logFFM, and with *p* taking the value of the regression coefficient to derive the index FM/FFM^*p*^. This method reduces the correlation between the index FM/FFM^*p*^ and the raw denominator (FFM), rendering the index both conceptually and statistically more robust [40]. Index values were small, and thus were multiplied by 1000 in order to increase the readability of results presented in tables.

We fitted a multiple regression model with FM/FFM^*p*^ as the dependent variable, and the following independent predictors: GDM status (coded 1 = GDM, 0 = NGT); mother’s age (continuous); ppBMI (continuous); GWG (continuous); type of delivery (1 = vaginal (reference), 2 = forceps, 3 = cesarean); newborn sex (1 = male, 0 = female); and gestational age (continuous). We fitted similar models for the GDM and NGT groups separately, where the dependent variable was FM/FFM^*p*^, and all independent variables were included except GDM status.

A full-model fit was initially employed for all models, including all candidate variables [41]. An assessment of goodness-of-fit based on multiple and adjusted R^2^ led us to drop non-significant variables and fit reduced models. The best-fit model using the full data set (GDM + NGT) was FM/FFM^*p*^ regressed on GDM, ppBMI, GWG, and newborn sex; for the GDM group it was FM/FFM^*p*^ on ppBMI and GWG; and for the NGT group it was FM/FFM^*p*^ on ppBMI, GWG and newborn sex. Because the variable %FM is commonly used by authors in similar analyses, models were repeated using %FM as the dependent variable, with results to be included as Supporting Information. We used the R package relaimpo (version 2.2–3) [42] to assess the relative importance of regressors, based on their contribution to total R^2^. The variance inflation factor was used to assess multicollinearity, and diagnostic plots and the Shapiro-Wilk test used to confirm normality of residuals. Significance tests were two-tailed at an alpha level of 0.05.

A number of observations were missing from the data set, particularly for the NGT subgroup. With the mice (Multivariate Imputation by Chained Equations) package (version 3.5.0) [43], multiple imputation was carried out on the full data set and the NGT-only data set, using the parameters m = 5, maxit = 100, seed = 500. ‘M’ is the number of multiple imputations, ‘maxit’ is the maximum number of iterations, and the value assigned to ‘seed’ is used to offset the random number generator [43]. As its name suggests, multiple imputation imputes missing values more than once, creating multiple predictions for each value and thus accounting for uncertainty in the imputations unlike e.g. mean imputation [44]. Full code for all analyses can be found at https://github.com/mkshirley87/GDM-Brazil.

### Ethics statement

The study was conducted according to the guidelines of the Declaration of Helsinki and all procedures involving human subjects were approved by the Ethics Committees of the School of Public Health, University of São Paulo, and the teaching hospital and maternity center Dr. Mário de Moraes Altenfelder Silva, where all data were collected (Approval Number 00749812.5.3001.0059). All mothers gave written informed consent for themselves and their newborns.

## Results

Seventy-two women with GDM and 211 with NGT were recruited. Characteristics of the mothers are shown in Table 1. GDM mothers were significantly older on average than NGT mothers, although the groups did not differ in parity; similar proportions of women in the GDM and NGT groups had more than one child. Proportions were also comparable between the groups for vaginal deliveries, although a higher proportion of GDM women had cesarean deliveries.

**Table 1:** Demographic, obstetric and socioeconomic characteristics of mothers with gestational diabetes mellitus (n = 72) and normal glucose tolerance (n = 211).

	Gestational diabetes mellitus (n = 72)		Normal glucose tolerance (n = 211)		
Variable	n (%)	Mean (SD) or Median (IQR)	n (%)	Mean (SD) or Median (IQR)	*p*
**Age (yrs)**^1^		29 (9.5)		25 (6.5)	<0.001
< 20	6 (8.5)		4 (1.9)		
20–25	12 (17)		113 (53.6)		
26–30	23 (32.4)		55 (26.1)		
31–35	13 (18.3)		25 (11.8)		
36–45	17 (24)		14 (6.6)		
**Height (m)**		1.60 (0.07)		1.60 (0.06)	0.95
**Parity**					0.93
1	25 (34.7)		76 (36.2)		
> 1	47 (65.3)		134 (63.8)		
**Type of delivery**					0.004
Vaginal	30 (41.7)		97 (47.3)		
Forceps	7 (9.7)		48 (23.4)		
Cesarean	35 (48.6)		60 (29.3)		
**Pre-pregnancy BMI (kg/m**^**2**^**)**^1^		30.1 (11.7)		24.2 (6.2)	<0.001
Underweight (< 18.5)	1 (1.4)		13 (6.5)		
Normal weight (18.5–24.99)	20 (27.8)		93 (46.7)		
Overweight/obese (≥ 25)	51 (70.8)		93 (46.7)		
**Gestational weight gain**^**2**^ **(kg)**^1^		11.4 (8.7)		11.0 (7.1)	0.24
Below recommendations	15 (20.8)		44 (28.6)		
Within recommendations	20 (27.8)		62 (40.3)		
Above recommendations	37 (51.4)		48 (31.2)		
**Work**					0.27
Yes	32 (44.4)		111 (53.0)		
No	40 (55.6)		99 (47.1)		
***Per capita* household income (Brazilian real)**^1^		416.7 (323.8)		400.0 (413.2)	0.85
> Minimum Brazilian wage	13 (19)		40 (22)		
< Minimum Brazilian wage	56 (81)		143 (78)		

IQR = interquartile range; SD = standard deviation; BMI = body mass index; minimum Brazilian wage in 2014 equivalent to R$724, or US$324, per month

^1^Data presented as median and IQR

^2^IOM (2009) guidelines [55]: recommended weight gain in pounds: 28–40 for BMI < 18.5 kg/m^2^, 25–35 for BMI 18.5–24.9 kg/m^2^, 15–25 for BMI 25.0–29.9 kg/m^2^, 11–20 for BMI ≥ 30 kg/m^2^

The groups were similar for average height and GWG, but GDM mothers had significantly higher ppBMI. Seventy-one percent of GDM mothers were overweight or obese, compared with 47% of NGT mothers. Around half of the mothers worked during pregnancy, and the majority reported a *per capita* household income below the minimum Brazilian wage, which at the time these data were collected was R$724 (US$324) per month.

Characteristics of the newborns are given in Table 2. The majority were born at term. Comparing newborns of GDM versus NGT mothers, there were no statistically significant differences in birth weight, birth length, FFM or head circumference, and proportions were similar for sex. In contrast, differences were observed for median FM and chest and abdominal circumferences.

**Table 2:** Gestational age, anthropometry and body composition of newborns of mothers with gestational diabetes mellitus (n = 72) and normal glucose tolerance (n = 211).

	Gestational diabetes mellitus (n = 72)		Normal glucose tolerance (n = 211)		
Variable	n (%)	Mean (SD) or Median (IQR)	n (%)	Mean (SD) or Median (IQR)	*p*
**Sex**					0.51
Female	35 (48.6)		113 (54.0)		
Male	37 (51.4)		96 (46.0)		
**Birth weight (g)**^1^		3425 (521.3)		3343 (516.3)	0.11
2500–3000	8 (11.1)		33 (15.9)		
3001–3500	32 (44.4)		110 (52.9)		
3501–4000	23 (31.9)		49 (23.6)		
4001–5000	9 (12.5)		15 (7.2)		
> 5000	0		1 (0.5)		
**Gestational age (wks)**^1^		39.0 (2.0)		39.0 (1.0)	0.03
36^2^ – 38	28 (38.9)		44 (21.2)		
39–40	38 (52.8)		138 (66.3)		
40–42	6 (8.3)		26 (12.5)		
**Fat mass (kg)**^1^		0.35 (0.3)		0.27 (0.2)	0.02
**Female**		0.35 (0.3)		0.32 (0.2)	
**Male**		0.34 (0.3)		0.24 (0.2)	
**Fat-free mass (kg)**		2.9 (0.3)		2.9 (0.3)	0.78
**Female**		2.8 (0.4)		2.8 (0.3)	
**Male**		3.0 (0.3)		3.0 (0.3)	
**Length (cm)**		49.1 (1.8)		49.3 (1.8)	0.46
**Female**		48.6 (1.7)		49.0 (1.9)	
**Male**		49.6 (1.8)		49.5 (1.5)	
**Head circumference (cm)**		34.8 (1.5)		34.7 (1.2)	0.66
**Female**		34.4 (1.5)		34.5 (1.3)	
**Male**		35.1 (1.4)		35.0 (1.1)	
**Chest circumference (cm)**		33.6 (1.7)		32.8 (1.6)	0.002
**Female**		33.2 (1.7)		32.8 (1.7)	
**Male**		33.9 (1.6)		32.8 (1.6)	
**Abdominal circumference (cm)**^1^		33.0 (2.0)		31.0 (2.4)	<0.001
**Female**		33.0 (2.9)		31.0 (2.5)	
**Male**		33.2 (1.6)		31.0 (2.1)	

IQR = interquartile range; SD = standard deviation

^1^Data presented as median and IQR

^2^Two infants in the NGT group had a gestational age of 36, and one in the diabetes group had a gestational age of 42

All missing observations (S1 Table) were successfully imputed. Following log-log regression analysis, the optimum index for infant FM in our sample was FM/FFM^1.4^. Shown in Table 3 are results of the multiple linear regression of FM/FFM^1.4^ on GDM status, ppBMI, GWG, and newborn sex. GWG, ppBMI and newborn sex were significantly predictive of infant FM controlling for one another and for GDM, which was not significant. The model explained 16% of the variance in newborn FM (multiple R^2^), with GDM contributing 1%, ppBMI 5%, GWG 3%, and sex 6%. Similar results were found using the imputed data set (also shown in Table 3). Iterations of the model which demonstrated a poorer fit to the data and were therefore rejected are given in S2 and S3 Tables.

**Table 3:** Results of multiple linear regression with neonatal FM/FFM^*p*^ as outcome, using the data set containing missing values, and following multiple imputation.

	Data set containing missing values			Data set following multiple imputation		
Predictor variable	Coefficient	95% CI	p	Coefficient	95% CI	p
Gestational diabetes mellitus (yes/no)	3.81	-7.32, 15.0	0.50	6.14	-4.35, 16.6	0.25
Pre-pregnancy BMI (kg/m^2^)	1.46	0.66, 2.27	<0.001	1.29	0.52, 2.08	0.001
Gestational weight gain (kg)	1.32	0.49, 2.15	0.002	1.15	0.29, 2.01	0.01
Male newborn sex	-17.8	-27.2, -8.29	<0.001	-17.1	-25.2, -9.03	<0.001
	Multiple R^2^ = 0.16; adjusted R^2^ = 0.15			Multiple R^2^ = 0.15; adjusted R^2^ = 0.14		

Table 4 shows results of the regression of FM/FFM^1.4^ on ppBMI and GWG, the best-fit model using the GDM-only data set. These two predictors explained 13% of the variance in the outcome (although adjusted R^2^ was slightly lower at 11%). Specifically, ppBMI explained 8% and GWG 5% of the variance. Given in Table 5 are results of FM/FFM^1.4^ regressed on ppBMI, GWG, and newborn sex in NGT mothers only. Newborn sex alone was significant, accounting for 11% of the variance in infant FM. Imputed models again yielded broadly similar results to those obtained with the original data sets containing missing observations. Rejected iterations of models in Tables 4 and 5 are given in S4–S6 Tables. Readers interested in model results with %FM can find these in S7–S10 Tables.

**Table 4:** Results of multiple linear regression for mothers with gestational diabetes mellitus (n = 72), with neonatal FM/FFM^*p*^ as outcome.

Predictor variable	Coefficient	95% CI	*p*
Pre-pregnancy BMI (kg/m^2^)	6.75	2.36, 11.1	0.003
Gestational weight gain (kg)	5.64	1.16, 10.1	0.014
Multiple R^2^ = 0.13; adjusted R^2^ = 0.11			

**Table 5:** Results of multiple linear regression for normal glucose tolerance mothers (n = 211) with neonatal FM/FFM^*p*^ as outcome, using the data set containing missing values, and following multiple imputation.

	Data set containing missing values			Data set following multiple imputation		
Predictor variable	Coefficient	95% CI	p	Coefficient	95% CI	p
Pre-pregnancy BMI (kg/m^2^)	0.58	0.01, 1.15	0.05	0.42	-0.07, 0.92	0.09
Gestational weight gain (kg)	0.54	-0.06, 1.15	0.08	0.35	-0.26, 0.96	0.25
Male newborn sex	-12.3	-18.3, -6.34	<0.001	-11.1	-16.2, -6.12	<0.001
	Multiple R^2^ = 0.15; adjusted R^2^ = 0.13			Multiple R^2^ = 0.11; adjusted R^2^ = 0.10		

## Discussion

Infants with increased BMI and FM are at greater risk of developing overweight and obesity in childhood and adult life [45–48], which in turn heightens risk of metabolic disorder [49, 50]. This contributes to epidemics of obesity and insulin resistance, as mothers with such conditions, including GDM [5], are more likely to give birth to infants with greater FM [16, 51, 52]. The current study identified significant associations of neonatal FM indexed by FM/FFM^1.4^ with maternal ppBMI, GWG and newborn sex, but not GDM status. Our combined (GDM + NGT) multiple regression model explained 16% of the variance in infant FM, which yielded an *f*^*2*^ value of 0.19 and is consistent with a medium effect size [53]. According to Cohen [53], *f*^*2*^ is the effect size index defined for squared multiple correlations (*R*^*2*^): the equation is *f*^*2*^ = *R*^*2*^/(1-*R*^*2*^).

Results for models run separately on GDM and NGT group data were somewhat different than those of the combined model. Here, we observed that ppBMI and GWG were predictive of newborn FM for GDM mothers (multiple R^2^ = 0.13), but not NGT mothers. The reverse was true for newborn sex, which was significant for the NGT (multiple R^2^ = 0.15), but not the GDM group. Considering the difference in average FM between the sexes in the NGT group (see Table 2), it is not surprising that sex was a strong predictor of the index FM/FFM^1.4^, where FM is assessed in relation to FFM. In general, it is well known that males and females exhibit differences in body composition from birth, although interestingly, the sexes are commonly more similar in FM whilst males demonstrate greater FFM than females [54]. Of course, this means that, on average, females at birth are proportionately fatter than males. In contrast to NGT newborns, males and females in the GDM group were more similar to one another in average FM, whilst results for FFM by sex in the GDM and NGT groups were virtually identical.

Our results for infant sex diverge from those of Lingwood et al. [20], who found that measures of maternal blood glucose were significantly predictive of FM in males (though not females), while mother’s BMI demonstrated an effect for females (but less so for males). However, our results are consistent with the hypothesis and results from a 2003 study by Catalano et al. [16], where significant differences were found between GDM and NGT groups for neonatal FM, but not FFM, length or birth weight. Similarly, in a recent systematic review and meta-analysis, Logan et al. [55] reported that FM of GDM infants, but not FFM, differed from NGT infants, and in fact in subgroup analyses this effect was significant only for males.

At the same time, Catalano et al. [16] found that maternal fasting glucose concentration correlated strongly with neonatal FM, while in our combined model, the variable denoting GDM status did not significantly predict FM. Maternal GDM status may be important for understanding variation in newborn FM, as suggested by the fact that ppBMI and GWG were significant predictors of newborn FM in our sample of GDM, but not NGT, women. However, more sensitive measures than the mere presence or absence of GDM, such as results of an oral glucose tolerance test, may be necessary to elucidate an association between GDM status and infant FM variation in the combined model. It is also possible that the women with GDM in our study had achieved good glycemic control, as they were recruited from a maternity care center which routinely takes steps to control diabetes in pregnancy. In a study carried out in Australia, for example, Au et al. [21] suggested good glycemic control as an explanation for a lack of difference in %FM of GDM versus NGT newborns. Glycemic control may have served to ameliorate adverse effects of GDM on levels of newborn FM in our sample, although a lack of data on our GDM mothers’ treatment and blood glucose levels means we are unable to explore this possibility further.

The portion of R-squared attributable to ppBMI and GWG was, respectively, 5% and 3% in the combined model, while the GDM group-specific model with ppBMI and GWG as the sole predictors explained 13% of the variance in newborn FM (a medium effect size [53]). A number of studies have demonstrated associations of ppBMI or GWG with newborn FM. For example, Josefson et al. [56] measured infant body composition by ADP, and found that mothers with excessive GWG (according to the 2009 Institute of Medicine (IOM) guidelines [57]) had infants with 50% greater FM than mothers who gained weight within the guidelines. The authors, however, only assessed mothers with a normal ppBMI. In our sample, a considerable proportion of women in both the GDM and NGT groups were overweight or obese according to ppBMI (71% and 41%, respectively). Following the IOM (2009) [57], 51% of GDM and 31% of NGT mothers exceeded GWG in relation to their ppBMI, although it should be noted that these guidelines were developed for women with NGT. No such recommendations exist for women with GDM. Indeed it appears, at least in non-GDM women, that the effect of GWG on infant FM may differ depending on whether women are normal weight, overweight or obese at the start of pregnancy [58], establishing along with other studies that ppBMI is a crucial factor for newborn FM [52, 59, 60].

GWG has further been associated with neonatal FM in studies involving both non-diabetic and diabetic pregnant women. For example, Starling et al. [61] observed a positive association of GWG with newborn %FM independent of ppBMI, while Blackwell et al. [23] found that excessive GWG was independently associated with increased FM in neonates of women with treated or untreated mild GDM. It has been suggested that GWG may in fact be a risk factor for GDM, with Gibson et al. [62] observing greater GWG up to 24 weeks’ gestation in women who ultimately developed GDM, although once again the results varied by ppBMI. In contrast, a meta-analysis indicated that excessive GWG may increase risk of GDM, irrespective of pre-ppBMI [63]. Beyond evidence for its contribution to GDM and increased newborn FM, excessive GWG has been associated with postpartum maternal FM retention [64], and weight, FM and insulin resistance of the offspring in childhood [65, 66].

Using ADP to measure newborn body composition, Au et al. [22] observed results similar to those we obtained with our combined regression model. Specifically, GWG, maternal ppBMI and sex, in addition to parity, ethnicity, gestational age, and smoking, explained 19% of the variance in newborn %FM in their cohort, whilst GDM status was not significant [22]. With respect to the association (or lack thereof) of GDM with offspring FM, one factor which could explain variation in findings across studies is whether or not researchers adjust for GWG. In two studies, Logan et al. [55, 67] identified positive associations between GDM and infant FM, however they did not control for GWG. Catalano et al. [16] found the association between GDM status and infant FM, already adjusted for several variables, attenuated considerably with further adjustment for maternal GWG. At the same time, study design and analysis may contribute to variation in findings. Results may vary depending on whether measures of maternal hyperglycemia are included in models along with GWG and additional potential confounders, versus models run separately for GDM and NGT mothers. Further work is required to disentangle the effects and interactions of ppBMI, GWG, and maternal glycemic status on offspring FM.

### Strengths and limitations

A main strength of this study is the use of ADP and the PEA POD system, a technique recognized for its high-quality measurement of infant body composition. Such measurements more accurately assess infant FM than e.g. BMI or skinfold thicknesses. Additionally, although our data set had several observations missing for the NGT subsample, we were able to carry out multiple imputation and demonstrate that results were largely unchanged when using the original or imputed data sets. We used FM/FFM^*p*^ as our index of newborn FM, which is considered more statistically robust than the oft-used %FM, and also better for assessing metabolic risk [40]. The FM/FFM^*p*^ index assesses FM in direct relation to FFM, which contains organs and metabolically-protective skeletal muscle [68–70].

Finally, this study recruited women in a South American country who were low-income, with the majority reporting a *per capita* household income less than the 2014 Brazilian minimum wage of R$724 (US$324). For comparison, the average *per capita* household income in São Paulo in the same year was R$1,432 (US$642) [71]. Although this may limit generalizability of the results, such populations require attention. Low-income women of Brazil, and South America more broadly, have so far been neglected in the literature on associations of maternal GDM, GWG and ppBMI with newborn FM.

Our study also has some important limitations. The sample size was relatively small, in particular for the GDM group. The GDM variable was binary, denoting women with or without GDM, rather than based on, for example, more specific measures of maternal glucose levels. At the same time, women were recruited when they gave birth, and no data were collected retrospectively on the method or degree of glycemic control in those with GDM. This left us unable to examine whether level of glycemic control might have contributed to a lack of association between GDM status and infant FM in our combined model. Additionally, despite its noted strengths, a limitation of the use of ADP is its reliance on sex- and age-specific assumed values for density and hydration of FFM, which may in fact demonstrate variation beyond these adjustments.

Similarly, despite the robustness of the FM/FFM^*p*^ index relative to %FM, the use of the index is arguably limited by the fact that it has yet to be widely adopted in the literature, and therefore it is more difficult to compare our results with prior work. If authors begin to adopt FM/FFM^*p*^ over e.g. %FM, it will help to identify a normative range against which future results can be compared. Finally, we were unable to assess the potential association of ethnic variation *within* the Brazilian population on our outcome of interest. This was untenable due to the high degree of heterogeneity in the population, and the fact that Brazilians are unlikely to consider themselves part of a specific ethnic or racial subgroup. Survey and study data typically query self-reported skin color, and it is unclear how responses map onto ethnicity or race in a meaningful way.

### Conclusions

In conclusion, this study identified increased FM relative to FFM in infants born to GDM versus NGT mothers, however GDM did not significantly predict infant FM in multiple regression models with adjustment for other factors. In contrast, ppBMI and GWG explained a portion of the variance in infant FM/FFM^1.4^ in both the combined model (8%) and GDM-specific model (13%), while sex of the newborn explained 11% of the variance in FM for the NGT group. The differing results of GDM versus NGT-group models suggest the potential importance of GDM status for understanding variation in neonatal FM, however our binary GDM variable may have precluded clearer results on this point. Going forward, prospective cohort studies are arguably needed which include ppBMI, GWG, and detailed data on the mother’s glycemic profile across pregnancy, including method and magnitude of glycemic control. Such studies would contribute to a greater understanding of the associations among these important maternal factors and their relative influence on offspring FM.

## Supporting information

S1 Table Missing data by variable for GDM and NGT mothers and newborns. (DOCX)

S2 Table Full model fit multiple linear regression with neonatal FM/FFM^*p*^ as outcome, using the data set with missing values, and following multiple imputation. (DOCX)

S3 Table Reduced multiple linear regression model with neonatal FM/FFM^*p*^ as outcome, using the data set with missing values, and following multiple imputation. (DOCX)

S4 Table Full model fit multiple linear regression for mothers with gestational diabetes mellitus (n = 72), with neonatal FM/FFM^*p*^ as outcome. (DOCX)

S5 Table Reduced multiple linear regression model for mothers with gestational diabetes mellitus (n = 72), with neonatal FM/FFM^*p*^ as outcome. (DOCX)

S6 Table Full model fit multiple linear regression for normal glucose tolerance mothers (n = 211) with neonatal FM/FFM^*p*^ as outcome, using the data set with missing values, and following multiple imputation. (DOCX)

S7 Table Percentage fat mass (%FM) of newborns of mothers with gestational diabetes mellitus (n = 72) and normal glucose tolerance (n = 211). (DOCX)

S8 Table Results of multiple linear regression in combined data set (GDM + NGT), with newborn %FM as outcome. (DOCX)

S9 Table Results of multiple linear regression for mothers with gestational diabetes mellitus (n = 72), with newborn %FM as outcome. (DOCX)

S10 Table Results of multiple linear regression for mothers with normal glucose tolerance (n = 211), with newborn %FM as outcome. (DOCX)

## References

[1] International Diabetes Federation. IDF Diabetes Atlas, 8th edn Brussels, Belgium: International Diabetes Federation, 2017 http://www.diabetesatlas.org.

[2] Zhu Y, Zhang C. Prevalence of gestational diabetes and risk of progression to type 2 diabetes: a global perspective. Curr Diab Rep. 2016;16: 7 10.1007/s11892-015-0699-x 26742932 PMC6675405

[3] Ferrara A. Increasing prevalence of gestational diabetes mellitus: a public health perspective. Diabetes Care. 2007;30: S141–S146. 10.2337/dc07-s206 17596462

[4] Chiefari E, Arcidiacono B, Foti D, Brunetti A. Gestational diabetes mellitus: an updated overview. J Endocrinol Invest. 2017;40: 899–909 10.1007/s40618-016-0607-5 28283913

[5] Piper LK, Stewart Z, Murphy HR. Gestational diabetes. Obstet Gynaecol Reprod Med. 2017;27: 171–176.

[6] Langer O, Yogev Y, Most O, Xenakis EMJ. Gestational diabetes: the consequences of not treating. Am J Obstet Gynecol. 2005;192: 989–997. 10.1016/j.ajog.2004.11.039 15846171

[7] Stuebe AM, Landon MB, Lai Y, Spong CY, Carpenter MW, Ramin SM, et al Maternal BMI, glucose tolerance and adverse pregnancy outcomes. Am J Obstet Gynecol. 2012;207: 62.e1–7. 22609018 10.1016/j.ajog.2012.04.035 PMC3482614

[8] NCD Risk Factor Collaboration. Worldwide trends in body-mass index, underweight, overweight and obesity from 1975 to 2016: a pooled analysis of 2416 population-based measurement studies in 128.9 million children, adolescents, and adults. Lancet. 2017;390: 2627–2642. 10.1016/S0140-6736(17)32129-3 29029897 PMC5735219

[9] Aspray TJ, Mugusi F, Rashid S, Whiting D, Edwards R, Alberti KG, et al Rural and urban differences in diabetes prevalence in Tanzania: the role of obesity, physical inactivity and urban living. Trans R Soc Trop Med Hyg. 2000;94: 637–644. 10.1016/s0035-9203(00)90216-5 11198647

[10] Shaw JE, Sicree RA, Zimmet PZ. Global estimates of the prevalence of diabetes for 2010 and 2030. Diabetes Res Clin Pract. 2010;87: 4–14. 10.1016/j.diabres.2009.10.007 19896746

[11] Buchanan TA, Xiang AH, Page KA. Gestational diabetes mellitus: risks and management during and after pregnancy. Nat Rev Endocrinol. 2012;8: 639–649. 10.1038/nrendo.2012.96 22751341 PMC4404707

[12] Goldenberg RL, McClure EM, Harrison MS, Miodovnik M. Diabetes during pregnancy in low- and middle-income countries. Am J Perinatol. 2016;33: 1227–1235. 10.1055/s-0036-1584152 27182990

[13] Bellamy L, Casas J-P, Hingorani AD, Williams D. Type 2 diabetes mellitus after gestational diabetes: a systematic review and meta-analysis. Lancet. 2009;373: 1773–1779. 10.1016/S0140-6736(09)60731-5 19465232

[14] World Health Organization. Global Report on Diabetes. Geneva, Switzerland: World Health Organization, 2016 https://www.who.int/diabetes/global-report/en/. 10.2337/db15-0956

[15] Chen L, Mayo R, Chatry A, Hu G. Gestational diabetes mellitus: its epidemiology and implication beyond pregnancy. Curr Epidemiol Rep. 2016;3: 1–11.

[16] Catalano PM, Thomas A, Huston-Presley L, Amini SB. Increased fetal adiposity: a very sensitive marker of abnormal in utero development. Am J Obstet Gynecol. 2003;189: 1698–1704. 10.1016/s0002-9378(03)00828-7 14710101

[17] Kubo A, Ferrara A, Windham GC, Greenspan LC, Deardorff J, Hiatt RA, et al Maternal hyperglycemia during pregnancy predicts adiposity of the offspring. Diabetes Care. 2014;37: 2996–3002. 10.2337/dc14-1438 25150158 PMC4207207

[18] Uebel K, Pusch K, Gedrich K, Schneider KTM, Hauner H, Bader BL. Effect of maternal obesity with and without gestational diabetes on offspring subcutaneous and preperitoneal adipose tissue development from birth up to year-1. BMC Pregnancy Childbirth. 2014;14: 138 10.1186/1471-2393-14-138 24720885 PMC4108007

[19] Nobile de Santis MS, Taricco E, Radaelli T, Spada E, Rigano S, Ferrazzi E, et al Growth of fetal lean mass and fetal fat mass in gestational diabetes. Ultrasound Obstet Gynecol. 2010;36: 328–337. 10.1002/uog.7575 20131333

[20] Lingwood BE, Henry AM, D’Emden MC, Fullerton A-M, Mortimer RH, Colditz PB, et al Determinants of body fat in infants of women with gestational diabetes mellitus differ with fetal sex. Diabetes Care. 2011;34: 2581–2585. 10.2337/dc11-0728 21994428 PMC3220854

[21] Au CP, Raynes-Greenow CH, Turner RM, Carberry AE, Jeffery HE. Body composition is normal in term infants born to mothers with well-controlled gestational diabetes mellitus. Diabetes Care. 2013a;36: 562–564. 23223404 10.2337/dc12-1557 PMC3579380

[22] Au CP, Raynes-Greenow CH, Turner RM, Carberry AE, Jeffery HE. Fetal and maternal factors associated with neonatal adiposity as measured by air-displacement plethysmography: a large cross-sectional study. Early Hum Dev. 2013b;89: 839–843. 23968962 10.1016/j.earlhumdev.2013.07.028

[23] Blackwell SC, Landon MB, Mele L, Reddy UM, Casey BM, Wapner RL, et al Relationship between excessive gestational weight gain and neonatal adiposity in women with mild gestational diabetes mellitus. Obstet Gynecol. 2016;128: 1325–1332. 10.1097/AOG.0000000000001773 27824768 PMC5123848

[24] Longmore DK, Barr ELM, Lee I-L, Barzi F, Kirkwood M, Whitbread C, et al Maternal body mass index, excess gestational weight gain, and diabetes are positively associated with neonatal adiposity in the Pregnancy and Neonatal Diabetes Outcomes in Remote Australia (PANDORA) study. Pediatr Obes. 2019;e12490 10.1111/ijpo.12490 30650263

[25] Yajnik CS, Fall CHD, Coyaji KJ, Hirve SS, Rao S, Barker DJP, et al Neonatal anthropometry: the thin-fat Indian baby. Int J Obes. 2003;27: 173–180. 10.1038/sj.ijo.802219 12586996

[26] Paley C, Hull H, Ji Y, Toro-Ramos T, Thornton J, Bauer J, et al Body fat differences by self-reported race/ethnicity in healthy term newborns. Pediatr Obes. 2016;11: 361–368. 10.1111/ijpo.12072 26509351 PMC4848178

[27] Toftemo I, Jenum AK, Lagerløv P, Júlíusson PB, Falk RS, Sletner L. Contrasting patterns of overweight and thinness among preschool children of different ethnic groups in Norway, and relations with maternal and early life factors. BMC Public Health. 2018;18: 1056 10.1186/s12889-018-5952-1 30139343 PMC6108110

[28] Rodrigo N, Glastras SJ. The emerging role of biomarkers in the diagnosis of gestational diabetes mellitus. J Clin Med. 2018;7: 120. 10.3390/jcm7060120 PMC6024961 29882903

[29] Lohman TG, Roche AF, Martorell R. Anthropometric standardization reference manual. Champaign, IL: Human Kinetics Books; 1988.

[30] Holland E, Moore Simas TA, Doyle Curiale DK, Liao X, Waring ME. Self-reported pre-pregnancy weight versus weight measured at first prenatal visit: effects on categorization of pre-pregnancy body mass index. Matern Child Health J. 2013;17: 10.1007/s10995-012-1210-9 23247668 PMC3622142

[31] Ismail LC, Bishop DC, Pang R, Ohuma EO, Kac G, Abrams B, et al Gestational weight gain standards based on women enrolled in the Fetal Growth Longitudinal Study of the INTERGROWTH-21^st^ Project: a prospective longitudinal cohort study. BMJ. 2016;352: i555 10.1136/bmj.i555 26926301 PMC4770850

[32] Ma G, Yao M, Liu Y, Lin A, Zou H, Urlando A, et al Validation of a new pediatric air-displacement plethysmograph for assessing body composition in infants. Am J Clin Nutr. 2004;79: 653–660. 10.1093/ajcn/79.4.653 15051611

[33] Ellis KJ, Yao M, Shypailo RJ, Urlando A, Wong WW, Heird WC. Body composition assessment in infancy: air-displacement plethysmography compared with a reference 4-compartment model. Am J Clin Nutr. 2007;85: 90–95. 10.1093/ajcn/85.1.90 17209182

[34] Andersen GS, Girma T, Wells JCK, Kœstel P, Leventi M, Hother A-L, et al Body composition from birth to 6 mo of age in Ethiopian infants: reference data obtained by air-displacement plethysmography. Am J Clin Nutr. 2013;98: 885–894. 10.3945/ajcn.113.063032 23985805

[35] Fomon SJ, Haschke F, Ziegler EE, Nelson SE. Body composition of reference children from birth to age 10 years. Am J Clin Nutr. 1982;35(suppl): 1169–1175. 7081099 10.1093/ajcn/35.5.1169

[36] R Core Team. R: a language and environment for statistical computing. Vienna, Austria: R Foundation for Statistical Computing, 2018 https://www.R-project.org/.

[37] Friis CM, Qvigstad E, Paasche Roland MC, Godang K, Voldner N, Bollerslev J, et al Newborn body fat: associations with maternal metabolic state and placental size. PLoS ONE. 2013;8: e57467 10.1371/journal.pone.0057467 23460863 PMC3583865

[38] Villar J, Puglia FA, Fenton TR, Ismail LC, Staines-Urias E, Giuliani F, et al Body composition at birth and its relationship with neonatal anthropometric ratios: the newborn body composition study of the INTERGROWTH-21^st^ project. Pediatr Res. 2017;82: 305–316. 10.1038/pr.2017.52 28445454 PMC5605677

[39] Breij LM, Kerkhof GF, de Lucia Rolfe E, Ong KK, Abrahamse-Berkeveld M, Hokken-Koelega ACS. Longitudinal fat mass and visceral fat during the first 6 months after birth in healthy infants: support for a critical window for adiposity in early life. Pediatr Obes. 2017;12: 286–294. 10.1111/ijpo.12139 27072083 PMC6186414

[40] Wells JCK, Victora CG. Indices of whole-body and central adiposity for evaluating the metabolic load of obesity. Int J Obes. 2005;29: 483–489. 10.1038/sj.ijo.0802899 15672103

[41] Sun G-W, Shook TL, Kay GL. Inappropriate use of bivariable analysis to screen risk factors for use in multivariable analysis. J Clin Epidemiol. 1996;49: 907–916. 8699212 10.1016/0895-4356(96)00025-x

[42] Grömping U. Relative importance for linear regression in R: the package relaimpo. J Stat Softw. 2006;17: 10.18637/jss.v017.i01.

[43] Van Buuren S, Groothuis-Oudshoorn. mice: Multivariate Imputation by Chained Equations in R. J Stat Softw. 2011;45: 1–67. https://www.jstatsoft.org/v45/i03/.

[44] Azur MJ, Stuart EA, Frangakis C, Leaf PJ. Multiple Imputation by Chained Equations: what is it and how does it work? Int J Methods Psychiatr Res. 2011;20: 40–49. 10.1002/mpr.329 21499542 PMC3074241

[45] Jouret B, Ahluwalia N, Cristini C, Dupuy M, Negre-Pages L, Grandjean H, et al Factors associated with overweight in preschool-age children in southwestern France. Am J Clin Nutr. 2007;85: 1643–1649. 10.1093/ajcn/85.6.1643 17556704

[46] Monasta L, Batty GD, Cattaneo A, Lutje V, Ronfani L, van Lenthe FJ, et al Early-life determinants of overweight and obesity: a review of systematic reviews. Obes Rev. 2010;11: 695–708. 10.1111/j.1467-789X.2010.00735.x 20331509

[47] Rios-Castillo I, Cerezo S, Corvalán C, Martínez M, Kain J. Risk factors during the prenatal period and the first year of life associated with overweight in 7-year-old low-income Chilean children. Matern Child Nutr. 2015;11: 595–605. 10.1111/mcn.12024 23241511 PMC6860292

[48] Admassu B, Wells JCK, Girma T, Belachew T, Ritz C, Owino V, et al Body composition during early infancy and its relation with body composition at 4 years of age in Jimma, an Ethiopian prospective cohort study. Nutr Diabetes. 2018;8: 46 10.1038/s41387-018-0056-7 30190452 PMC6127223

[49] Biro FM, Wien M. Childhood obesity and adult morbidities. Am J Clin Nutr. 2010;91(suppl): 1499S–1505S. 20335542 10.3945/ajcn.2010.28701B PMC2854915

[50] Rocha Sentalin PB, Oliveira Pinheiro A, Oliveira RR, Zângaro RA, Aparecida Campos L, Baltatu OC. Obesity and metabolic syndrome in children in Brazil: the challenge of lifestyle change. Medicine. 2019;98: 19. 10.1097/MD.0000000000015666 PMC6531096 31083270

[51] Dabelea D, Crume T. Maternal environment and the transgenerational cycle of obesity and diabetes. Diabetes. 2011;60: 1849–1855. 10.2337/db11-0400 21709280 PMC3121421

[52] Catalano PM, McIntyre HD, Cruickshank JK, McCance DR, Dyer AR, Metzger BE, et al The Hyperglycemia and Adverse Pregnancy Outcome study: associations of GDM and obesity with pregnancy outcomes. Diabetes Care. 2012;35: 780–786. 10.2337/dc11-1790 22357187 PMC3308300

[53] Cohen J. A power primer. Psychol Bull. 1992;112: 155–159. 19565683 10.1037//0033-2909.112.1.155

[54] Wells JCK. Sexual dimorphism of body composition. Best Pract Res Clin Endocrinol Metab. 2007;21: 415–430. 10.1016/j.beem.2007.04.007 17875489

[55] Logan KM, Gale C, Hyde MJ, Santhakumaran S, Modi N. Diabetes in pregnancy and infant adiposity: systematic review and meta-analysis. Arch Dis Child Fetal Neonatal Ed. 2017;102: F65–F72. 10.1136/archdischild-2015-309750 27231266 PMC5256410

[56] Josefson JL, Hoffmann JA, Metzger BE. Excessive weight gain in women with a normal pre-pregnancy BMI is associated with increased neonatal adiposity. Pediatr Obes. 2013;8: e33–e36. 10.1111/j.2047-6310.2012.00132.x 23283756 PMC4076951

[57] Institute of Medicine. Weight Gain during Pregnancy: Reexamining the Guidelines. Washington, DC: National Academies Press; 2009. 20669500

[58] Waters TP, Huston-Presley L, Catalano PM. Neonatal body composition according to the revised Institute of Medicine recommendations for maternal weight gain. J Clin Endocrinol Metab. 2012;97: 3648–3654. 10.1210/jc.2012-1781 22821895 PMC3674292

[59] Hull HR, Thornton JC, Ji Y, Paley C, Rosenn B, Mathews P, et al Higher infant body fat with excessive gestational weight gain in overweight women. Am J Obstet Gynecol. 2011;205: 211.e1–7. 21621185 10.1016/j.ajog.2011.04.004 PMC3170486

[60] Breij LM, Steegers-Theunissen RPM, Briceno D, Hokken-Koelega ACS. Maternal and fetal determinants of neonatal body composition. Horm Res Pediatr. 2015;84: 388–395. 10.1159/000441298 26492188

[61] Starling AP, Brinton JT, Glueck DH, Shapiro AL, Harrod CS, Lynch AM, et al Associations of maternal BMI and gestational weight gain with neonatal adiposity in the Healthy Start study. Am J Clin Nutr. 2015;101: 302–309. 10.3945/ajcn.114.094946 25646327 PMC4307203

[62] Gibson KS, Waters TP, Catalano PM. Maternal weight gain in women who develop gestational diabetes mellitus. Obstet Gynecol. 2012;119: 560–565. 10.1097/AOG.0b013e31824758e0 22353954

[63] Brunner S, Stecher L, Ziebarth S, Nehring I, Rifas-Shiman SL, Sommer C, et al Excessive gestational weight gain prior to glucose screening and the risk of gestational diabetes mellitus: a meta-analysis. 10.1007/s00125-015-3686-5 26141788

[64] Butte NF, Ellis KJ, Wong WW, Hopkinson JM, O’Brien Smith E. Composition of gestational weight gain impacts maternal fat retention and infant birth weight. Am J Obstet Gynecol. 2003;189: 1423–1432. 10.1067/s0002-9378(03)00596-9 14634581

[65] Ensenauer R, Chmitorz A, Riedel C, Fenske N, Hauner H, Nennstiel-Ratzel U, et al Effects of suboptimal or excessive gestational weight gain on childhood overweight and abdominal adiposity: results from a retrospective cohort study. Int J Obes. 2013;37: 505–512. 10.1038/ijo.2012.226 23357957

[66] Tam CHT, Ma RCW, Yuen LY, Ozaki R, Li AM, Hou Y, et al The impact of maternal gestational weight gain on cardiometabolic risk factors in children. Diabetologia. 2018;61: 2539–2548. 10.1007/s00125-018-4724-x 30225524 PMC6223878

[67] Logan KM, Emsley RJ, Jeffries S, Andrzejewska I, Hyde MJ, Gale C, et al Development of early adiposity in infants of mothers with gestational diabetes mellitus. Diabetes Care. 2016; 10.2337/dc16-0030 27208326

[68] Bayol SA, Bruce CR, Wadley GD. Growing healthy muscles to optimise metabolic health into adult life. J Dev Orig Health Dis. 2014;5: 420–434. 10.1017/S2040174414000452 25296864

[69] Srikanthan P, Karlamangla AS. Relative muscle mass is inversely associated with insulin resistance and prediabetes: findings from the third national health and nutrition examination survey. J Clin Endocrinol Metab. 2011;96: 2898–2903. 10.1210/jc.2011-0435 21778224

[70] Lee MJ, Kim E-H, Bae S-J, Choe J, Jung CH, Lee WJ, et al Protective role of skeletal muscle mass against progression from metabolically healthy to unhealthy phenotype. Clin Endocrinol. 2019;90: 102–113. 10.1111/cen.13874 30290006

[71] Diretoria de Pesquisas, Coordenação de Trabalho e Rendimento, Pesquisa Nacional por Amostra de Domicílios Contínua; 2014 [cited 2019 June 26]. Database: IBGE [Internet]. Available from: http://www.ibge.gov.br/estatisticas/sociais/rendimento-despesa-e-consumo/9171-pesquisa-nacional-por-amostra-de-domicilios-continua-mensal.html?edicao=20652&t=downloads.

