# Supplementary material for: Gestational diabetes mellitus, pre-pregnancy body mass index, and gestational weight gain as risk factors for increased fat mass in Brazilian newborns
Source: PLoS One. 2019 Aug 29;14(8):e0221971. doi: 10.1371/journal.pone.0221971 (PMC6715169; doi:10.1371/journal.pone.0221971)
Supplement: S1 Table — (DOCX) [file pone.0221971.s001.docx]

**S1 Table. Missing data by variable for GDM and NGT mothers and newborns.**

| **Variable name** | **Number of missing observations (GDM)** | **Number of missing observations (NGT)** |
| --- | --- | --- |
| Mother’s age | 1 | 0 |
| Parity | 0 | 1 |
| Type of delivery | 0 | 6 |
| Pre-pregnancy BMI | 0 | 12 |
| Gestational weight gain | 0 | 49 |
| Work | 0 | 1 |
| *Per capita* income | 3 | 28 |
| Newborn sex | 0 | 2 |
| Birth weight | 0 | 3 |
| Gestational age | 0 | 3 |
| Newborn length | 0 | 4 |
| Newborn head circumference | 0 | 14 |
| Newborn chest circumference | 0 | 21 |
| Newborn abdominal circumference | 0 | 18 |
