# Supplementary material for: Gestational diabetes mellitus, pre-pregnancy body mass index, and gestational weight gain as risk factors for increased fat mass in Brazilian newborns
Source: PLoS One. 2019 Aug 29;14(8):e0221971. doi: 10.1371/journal.pone.0221971 (PMC6715169; doi:10.1371/journal.pone.0221971)
Supplement: S2 Table — (DOCX) [file pone.0221971.s002.docx]

**S2 Table**. **Full model fit multiple linear regression with neonatal FM/FFM*^p^* as outcome, using the data set with missing values, and following multiple imputation.**

|  | Data set with missing values | | | Data set following multiple imputation | | |
| --- | --- | --- | --- | --- | --- | --- |
| **Predictor variable** | **Coefficient** | **95% CI** | **p** | **Coefficient** | **95% CI** | **p** |
| Gestational diabetes mellitus (yes/no) | 1.58 | -10.2, 13.3 | 0.79 | 4.86 | -5.93, 15.7 | 0.38 |
| Mother’s age (yrs) | 0.18 | -0.65, 1.01 | 0.67 | 0.20 | -0.54, 0.94 | 0.59 |
| Pre-pregnancy BMI (kg/m^2^) | 1.35 | 0.50, 2.19 | 0.002 | 1.22 | 0.39, 2.04 | 0.004 |
| Gestational weight gain (kg) | 1.32 | 0.48, 2.17 | 0.002 | 1.19 | 0.30, 2.08 | 0.010 |
| Forceps delivery^1^ | -11.7 | -24.9, 1.55 | 0.08 | -8.72 | -20.1, 2.66 | 0.13 |
| Cesarean delivery^1^ | 0.14 | -10.9, 11.2 | 0.98 | -2.48 | -12.4, 7.42 | 0.62 |
| Male newborn sex | -17.0 | -26.9, -7.14 | <0.001 | -15.9 | -24.2, -7.70 | <0.001 |
| Gestational age (wks) | -0.65 | -5.13, 3.84 | 0.78 | -0.31 | -3.97, 3.36 | 0.87 |
|  | Multiple R^2^= 0.18; adjusted R^2^= 0.15 | | | Multiple R^2^= 0.16; adjusted R^2^= 0.14 | | |

^1^Dummy-coded ‘type of delivery’ variable, with vaginal delivery as reference
