# Supplementary material for: Gestational diabetes mellitus, pre-pregnancy body mass index, and gestational weight gain as risk factors for increased fat mass in Brazilian newborns
Source: PLoS One. 2019 Aug 29;14(8):e0221971. doi: 10.1371/journal.pone.0221971 (PMC6715169; doi:10.1371/journal.pone.0221971)
Supplement: S3 Table — (DOCX) [file pone.0221971.s003.docx]

**S3 Table. Reduced multiple linear regression model with neonatal FM/FFM*^p^* as outcome, using the data set with missing values, and following multiple imputation.**

|  | Data set with missing values | | | Data set following multiple imputation | | |
| --- | --- | --- | --- | --- | --- | --- |
| **Predictor variable** | **Coefficient** | **95% CI** | **p** | **Coefficient** | **95% CI** | **p** |
| Gestational diabetes mellitus (yes/no) | 1.87 | -9.42, 13.2 | 0.74 | 5.55 | -4.99, 16.1 | 0.30 |
| Pre-pregnancy BMI (kg/m^2^) | 1.40 | 0.57, 2.22 | 0.001 | 1.25 | 0.43, 2.06 | 0.003 |
| Gestational weight gain (kg) | 1.32 | 0.48, 2.16 | 0.002 | 1.19 | 0.31, 2.08 | 0.009 |
| Forceps delivery^1^ | -11.8 | -24.8, 1.15 | 0.07 | -9.11 | -20.4, 2.17 | 0.11 |
| Cesarean delivery^1^ | -0.11 | -11.0, 10.8 | 0.98 | -2.54 | -12.4, 7.32 | 0.61 |
| Male newborn sex | -16.5 | -26.2, -6.88 | <0.001 | -16.2 | -24.3, -7.96 | <0.001 |
|  | Multiple R^2^= 0.18; adjusted R^2^= 0.15 | | | Multiple R^2^= 0.16; adjusted R^2^= 0.14 | | |

^1^Dummy-coded ‘type of delivery’ variable, with vaginal delivery as reference
