# Supplementary material for: Gestational diabetes mellitus, pre-pregnancy body mass index, and gestational weight gain as risk factors for increased fat mass in Brazilian newborns
Source: PLoS One. 2019 Aug 29;14(8):e0221971. doi: 10.1371/journal.pone.0221971 (PMC6715169; doi:10.1371/journal.pone.0221971)
Supplement: S4 Table — (DOCX) [file pone.0221971.s004.docx]

**S4 Table. Full model fit multiple linear regression for mothers with gestational diabetes mellitus (n = 72), with neonatal FM/FFM*^p^* as outcome.**

| **Predictor variable** | **Coefficient** | **95% CI** | ***p*** |
| --- | --- | --- | --- |
| Mother’s age (yrs) | 0.45 | -4.43, 5.32 | 0.85 |
| Pre-pregnancy BMI (kg/m^2^) | 6.21 | 1.36, 11.1 | 0.013 |
| Gestational weight gain (kg) | 5.55 | 0.71, 10.4 | 0.025 |
| Type of delivery^1^ | 2.14 | -33.3, 37.6 | 0.90 |
| Male newborn sex | -19.2 | -87.4, 49.1 | 0.58 |
| Gestational age (wks) | -5.59 | -31.4, 20.3 | 0.67 |
| Multiple R^2^ = 0.15; adjusted R^2^ = 0.07 | | | |

^1^Vaginal (reference), forceps, cesarean
