# Supplementary material for: Gestational diabetes mellitus, pre-pregnancy body mass index, and gestational weight gain as risk factors for increased fat mass in Brazilian newborns
Source: PLoS One. 2019 Aug 29;14(8):e0221971. doi: 10.1371/journal.pone.0221971 (PMC6715169; doi:10.1371/journal.pone.0221971)
Supplement: S5 Table — (DOCX) [file pone.0221971.s005.docx]

**S5 Table. Reduced multiple linear regression model for mothers with gestational diabetes mellitus (n = 72), with neonatal FM/FFM*^p^* as outcome.**

| **Predictor variable** | **Coefficient** | **95% CI** | ***p*** |
| --- | --- | --- | --- |
| Pre-pregnancy BMI (kg/m^2^) | 6.43 | 1.87, 11.0 | 0.006 |
| Gestational weight gain (kg) | 5.56 | 1.05, 10.1 | 0.017 |
| Male newborn sex | -18.2 | -81.9, 45.4 | 0.57 |
| Multiple R^2^ = 0.14; adjusted R^2^ = 0.10 | | | |
