# Supplementary material for: Gestational diabetes mellitus, pre-pregnancy body mass index, and gestational weight gain as risk factors for increased fat mass in Brazilian newborns
Source: PLoS One. 2019 Aug 29;14(8):e0221971. doi: 10.1371/journal.pone.0221971 (PMC6715169; doi:10.1371/journal.pone.0221971)
Supplement: S6 Table — (DOCX) [file pone.0221971.s006.docx]

**S6 Table. Full model fit multiple linear regression for normal glucose tolerance mothers (n = 211) with neonatal FM/FFM*^p^* as outcome, using the data set with missing values, and following multiple imputation.**

|  | Data set with missing values | | | Data set following multiple imputation | | |
| --- | --- | --- | --- | --- | --- | --- |
| **Predictor variable** | **Coefficient** | **95% CI** | **p** | **Coefficient** | **95% CI** | **p** |
| Mother’s age (yrs) | 0.12 | -0.47, 0.70 | 0.69 | 0.11 | -0.38, 0.61 | 0.66 |
| Pre-pregnancy BMI (kg/m^2^) | 0.45 | -0.17, 1.06 | 0.16 | 0.32 | -0.21, 0.85 | 0.24 |
| Gestational weight gain (kg) | 0.54 | -0.07, 1.16 | 0.08 | 0.39 | -0.29, 1.06 | 0.25 |
| Forceps delivery^1^ | -6.04 | -13.9, 1.81 | 0.13 | -4.55 | -11.1, 2.02 | 0.17 |
| Cesarean delivery^1^ | 0.65 | -6.59, 7.89 | 0.86 | -1.02 | -7.16, 5.11 | 0.74 |
| Male newborn sex | -12.1 | -18.4, -5.82 | <0.001 | -10.5 | -15.6, -5.40 | <0.001 |
| Gestational age (wks) | 1.47 | -1.71, 4.65 | 0.36 | 0.68 | -1.76, 3.11 | 0.58 |
|  | Multiple R^2^= 0.18; adjusted R^2^= 0.14 | | | Multiple R^2^= 0.12; adjusted R^2^= 0.09 | | |

^1^Dummy-coded ‘type of delivery’ variable, with vaginal delivery as reference
