# Supplementary material for: Gestational diabetes mellitus, pre-pregnancy body mass index, and gestational weight gain as risk factors for increased fat mass in Brazilian newborns
Source: PLoS One. 2019 Aug 29;14(8):e0221971. doi: 10.1371/journal.pone.0221971 (PMC6715169; doi:10.1371/journal.pone.0221971)
Supplement: S7 Table — (DOCX) [file pone.0221971.s007.docx]

**S7 Table**. **Percentage fat mass (%FM) of newborns of mothers with gestational diabetes mellitus (n = 72) and normal glucose tolerance (n = 211)**.

|  | Gestational diabetes mellitus (n = 72) | Normal glucose tolerance (n = 211) |  |
| --- | --- | --- | --- |
|  | **Median (IQR)** | **Median (IQR)** | ***p*** |
| **%Fat mass** | 10.4 (8.0) | 8.7 (6.2) | 0.02 |
| Female | 11.2 (7.5) | 10.2 (6.0) |  |
| Male | 10.2 (7.0) | 7.5 (5.7) |  |
