# Supplementary material for: Gestational diabetes mellitus, pre-pregnancy body mass index, and gestational weight gain as risk factors for increased fat mass in Brazilian newborns
Source: PLoS One. 2019 Aug 29;14(8):e0221971. doi: 10.1371/journal.pone.0221971 (PMC6715169; doi:10.1371/journal.pone.0221971)
Supplement: S8 Table — (DOCX) [file pone.0221971.s008.docx]

**S8 Table. Results of multiple linear regression in combined data set (GDM + NGT), with newborn %FM as outcome**.

| **Predictor variable** | **Coefficient** | **95% CI** | ***p*** |
| --- | --- | --- | --- |
| Gestational diabetes mellitus (yes/no) | 0.31 | -1.06, 1.68 | 0.66 |
| Pre-pregnancy BMI (kg/m^2^) | 0.18 | 0.08, 0.28 | <0.001 |
| Gestational weight gain (kg) | 0.16 | 0.06, 0.27 | 0.002 |
| Male newborn sex | -1.94 | -3.10, -0.77 | 0.001 |
| Multiple R^2^ = 0.15; adjusted R^2^ = 0.13 | | | |
