# Supplementary material for: Gestational diabetes mellitus, pre-pregnancy body mass index, and gestational weight gain as risk factors for increased fat mass in Brazilian newborns
Source: PLoS One. 2019 Aug 29;14(8):e0221971. doi: 10.1371/journal.pone.0221971 (PMC6715169; doi:10.1371/journal.pone.0221971)
Supplement: S10 Table — (DOCX) [file pone.0221971.s010.docx]

**S10 Table. Results of multiple linear regression for mothers with normal glucose tolerance (n = 211), with newborn %FM as outcome.**

| **Predictor variable** | **Coefficient** | **95% CI** | ***p*** |
| --- | --- | --- | --- |
| Pre-pregnancy BMI (kg/m^2^) | 0.16 | 0.03, 0.29 | 0.016 |
| Gestational weight gain (kg) | 0.15 | 0.01, 0.29 | 0.034 |
| Male newborn sex | -2.25 | -3.63, -0.86 | 0.002 |
| Multiple R^2^ = 0.13; adjusted R^2^ = 0.11 | | | |
